# Effects of Alkali Modulus on Early-Age Performance and Hydration Mechanisms of Slag–Phosphogypsum Composite Alkali-Activated Materials

**DOI:** 10.3390/ma19030459

**Published:** 2026-01-23

**Authors:** Xushuai Qin, Min Li, Mengzhang Chen, Chunxue Wang, Shenghan Zhuang, Zhanfang Huang, Jiaolong Ren

**Affiliations:** 1 School of Civil Engineering and Geomatics, Shandong University of Technology, Zibo 255000, China; qinxushuai9185@163.com (X.Q.); 23507030882@stumail.sdut.edu.cn (M.C.); 24507030899@stumail.sdut.edu.cn (C.W.); huangzhanfang@163.com (Z.H.); 2 School of Transportation and Vehicle Engineering, Shandong University of Technology, Zibo 255000, China; 22507020009@stumail.sdut.edu.cn; 3 Department of Bridge Engineering, Southwest Jiaotong University, Chengdu 610031, China; shenghanzhuang@my.swjtu.edu.cn

**Keywords:** slag–phosphogypsum composite alkali-activated materials, alkali modulus, mechanical property, hydration mechanism

## Abstract

The disposal of phosphogypsum has emerged as a significant challenge for the phosphorus chemical industry in China in recent years. Utilizing phosphogypsum in alkali-activated materials represents an effective approach to valorize this byproduct. The alkali modulus is a critical parameter affecting the performance characteristics of phosphogypsum-based alkali-activated materials. This study aims to investigate the effects of the alkali modulus on the early-age properties (setting time, fluidity, flexural strength, and compressive strength) and hydration mechanisms of slag–phosphogypsum composite alkali-activated materials (HSFP) across various slag–phosphogypsum–fly ash systems, thereby identifying the optimal alkali modulus. The findings demonstrate that an alkali modulus of 1.35 optimally enhances the mechanical performance of HSFP. At this specific modulus, the equilibrium between alkalinity and soluble silica availability facilitates complete hydration, resulting in a dense gel-crystal microstructure characterized by the highest C-(A)-S-H gel content (58.2%) after 28 days. The effect of the alkali modulus on mechanical properties is contingent upon the fly ash-to-phosphogypsum (FA:PG) ratio, whereas its effect on fluidity and setting time is negligible. The effect of alkali modulus on the strength of HSFP is significantly affected by the fly ash-to-phosphogypsum (FA:PG) ratio. At an FA:PG ratio of 4:6, the flexural strength initially decreases and then increases as the alkali modulus values increase, while the compressive strength shows a consistent upward trend. At FA:PG ratios of 1:5 and 1:9, the flexural strength increases linearly with the alkali modulus, whereas the compressive strength first rises and then experiences a slight decline. These results offer both theoretical insights and practical guidance for the optimization of phosphogypsum-based cementitious material formulations, thereby supporting their potential for large-scale application.

## 1. Introduction

Phosphogypsum is a solid byproduct generated during the wet process of phosphoric acid production. Currently, the total accumulation of phosphogypsum in China exceeds 90 million tons, yet its comprehensive utilization rate remains below 5%. The management and disposal of phosphogypsum have emerged as significant challenges for the phosphorus chemical industry in China in recent years. Unregulated discharge and accumulation of this waste pose severe threats to the ecological environment, leading to groundwater contamination and inefficient land use. Utilizing phosphogypsum as an alkaline-activated grouting material represents an effective strategy for resource recovery [1]. Alkali-activated materials are a novel class of civil engineering materials that have developed rapidly in recent years. These materials are typically synthesized from precursors such as slag, fly ash, and gypsum, combined with alkaline activators including calcium oxide and sodium hydroxide. The production process of alkali-activated materials does not require calcination, resulting in carbon dioxide emissions that are approximately 20% of those associated with traditional cement-based materials, thereby providing substantial environmental benefits [2,3].

In recent years, considerable scholarly attention has been directed toward the development of cementitious materials derived from phosphogypsum. Notable studies by Chen et al. [4], Deng et al. [5], You et al. [6], Liu et al. [7], Wang et al. [8], Ren et al. [9], and Wang et al. [10] have reported the synthesis of phosphogypsum-based cementitious composites through the incorporation of additives such as slag, fly ash, and other supplementary materials. These innovations not only enabled the stabilization and effective utilization of phosphogypsum but also advanced sustainable, low-carbon practices within the construction industry. Nonetheless, the use of phosphogypsum as a precursor in alkali-activated materials might decrease the initial mechanical strength due to the generation of expansive degradation products. Furthermore, the long-term durability of these materials under extreme environmental conditions has yet to be comprehensively evaluated [11]. Qu et al. [12] conducted a comprehensive evaluation of phosphogypsum pretreatment techniques combined with auxiliary cementitious materials, examining their effect on workability, mechanical properties, shrinkage behavior, and transport characteristics of the resulting composites. Li et al. [13] proposed an environmentally benign ultra-low-alkalinity cementitious material composed of phosphogypsum, granulated fine slag, and sulfoaluminate cement, which demonstrates rapid setting, reduced hydration heat, improved mortar strength, and effective immobilization of hazardous constituents inherent in phosphogypsum. Tang et al. [14] developed a cementitious material derived from solid waste by blending phosphogypsum with granulated blast furnace slag and fly ash, systematically investigating its mechanical performance, hydration mechanisms, and pollutant stabilization capabilities. In a similar vein, Meng et al. [15] engineered a high-performance composite cementitious material entirely from solid wastes, offering an innovative strategy for the co-disposal and resource recovery of diverse solid waste streams. Hua et al. [16] examined a cementitious system primarily composed of phosphogypsum and finely ground blast furnace slag, assessing its effect resistance, flexural strength, freeze-thaw durability, and microstructural features. Shi et al. [17] prepared environmentally friendly cementitious materials with high proportions of phosphogypsum and hydrated lime, analyzing the effects of raw material ratios on mechanical strength. Furthermore, Yang et al. [18] formulated backfilling cementitious materials by combining phosphogypsum with slag, utilizing orthogonal experimental designs to optimize material strength across varying proportions and fineness levels, thereby identifying the optimal composition for phosphogypsum-based cementitious applications. These studies found that alkaline activation constitutes a pivotal factor affecting the performance of phosphogypsum-based cementitious materials [19]. Zhang et al. [20] incorporated slag and fly ash into hemihydrate phosphogypsum and employed NaOH as an alkaline activator to examine its effect on the mechanical properties of the resulting cementitious composites. Similarly, Ouyang et al. [21] investigated the effect of steel slag powder as an alkaline activator on the hydration behavior and mechanical strength of sulfate phosphogypsum slag cement. Ma et al. [22] developed a steel fiber-reinforced super-sulfate cement by combining phosphogypsum, finely ground blast furnace slag, and steel slag, supplemented with alkaline activators such as NaOH, to evaluate its mechanical performance. Lin et al. [23] formulated composite cementitious materials based on phosphogypsum and slag powder, augmented with quicklime, NaOH, and NaHCO_3_, to assess the effects of various admixtures on fluidity, setting time, and compressive strength. Peng et al. [24] explored the effect of different alkalinity levels on the strength and other properties of hemihydrate phosphogypsum-based cementitious materials. In a related investigation, Lin et al. [25] examined the effect of varying lime content on the mechanical properties and volumetric expansion of phosphogypsum-based cementitious materials, emphasizing the critical role of alkaline environments in the formation and distribution of alunite. Pan et al. [26] prepared phosphogypsum-based cementitious slurry backfill materials using phosphogypsum and fly ash as primary raw materials, with red mud serving as an alkaline activator, and analyzed their mechanical characteristics. Sun et al. [27] assessed the effects of different types and dosages of alkaline materials on impurity content and expansion behavior within phosphogypsum-based cementitious systems. Wang et al. [28] evaluated the effect of five distinct alkaline activator combinations on the strength of a novel phosphogypsum-based geopolymer cementitious material. Peng et al. [29] proposed an innovative all-solid-waste cementitious material by combining modified phosphogypsum with finely ground blast furnace slag and steel slag, utilizing sodium hydroxide as the alkaline activator to investigate its strength development. Although these studies have clarified the effects of varying alkali dosages on mechanical properties, there remains a paucity of comprehensive and systematic research specifically addressing the role of alkaline modulus in these materials.

Among the multiple factors affecting the performance of phosphogypsum-based cementitious materials, the alkali modulus of the alkali activator emerges as a critical parameter governing their behavior [30,31]. However, most existing studies primarily focus on the effects of individual alkali activator parameters, lacking a comprehensive evaluation of the synergistic interactions between alkali dosage and alkali modulus [32]. This gap impedes a thorough understanding of the interactive mechanisms through which these factors affect hydration kinetics and microstructural evolution. For instance, in the development of geopolymer formulations utilizing the Design of Experiments methodology, it has been demonstrated that interactions between alkali-related parameters and other variables—such as precursor ratios and curing regimes—significantly affect material performance. This finding underscores the necessity for systematic optimization of these parameters. Additionally, distinct precursor combinations demand specific alkali modulus values, emphasizing the need for targeted investigations tailored to particular mix designs [33]. Moreover, the current literature reveals a notable gap regarding the adaptability of alkali parameters within diverse slag–phosphogypsum–fly ash blended systems, with limited attention given to the interactive effects arising from variations in substrate proportions and alkali activator characteristics [34].

Addressing these challenges, this study focuses on the alkaline modulus as a key regulatory factor, systematically evaluating its effect on the early-age properties of slag–phosphogypsum composite alkali-activated materials (HSFP) across various slag–phosphogypsum–fly ash composite systems. The synergistic effect of the alkaline modulus on the formation of hydration products is also investigated by microscopic characterization methods, such as X-ray diffraction (XRD), scanning electron microscopy (SEM), and thermogravimetric-differential scanning calorimetry (TG-DSC). The results identify optimal conditions for enhancing mechanical properties, thereby providing both a theoretical framework and practical recommendations for the optimization of mix designs and facilitating the large-scale utilization of phosphogypsum-based cementitious materials.

## 2. Materials and Methods

The selection of various precursors and alkali modulus exerts a considerable effect on performance results. Consequently, this section offers an in-depth examination of the physical characteristics of the materials utilized in the experiments, accompanied by a thorough account of the experimental methodologies implemented.

### 2.1. Materials

#### 2.1.1. Precursor

(1) S95 Grade Slag Powder

The present study employed S95-grade slag powder sourced from Longze Water Purification Materials Co., Ltd., Gongyi, China. The slag powder demonstrated an activity index of 84.2% at 7 days and 98.5% at 28 days. Comprehensive performance data are presented in Table 1 and Table 2.

(2) Fly Ash

The fly ash utilized in this investigation was sourced from Boheng Mineral Products Trading Co., Ltd., Lingshou, China. The material exhibited a particle size distribution equivalent to 400 mesh. Detailed characteristics of fly ash are presented in Table 3 and Table 4.

(3) Phosphogypsum

Phosphogypsum has a light gray, powdery texture. Its performance parameters are detailed in Table 5.

(4) Ca(OH)_2_

The calcium hydroxide employed in this investigation was procured from Jinan Xinkaiming Chemical Co., Ltd., Jinan, China. It has a density of 2.24 g/cm^3^, a loss on ignition value of 22.52%, and a moisture content of 0.23%. The chemical composition and associated concentrations are presented in Table 6.

#### 2.1.2. Alkali Activator

(1) Na_2_SiO_3_ (Water Glass)

The sodium silicate employed in this investigation was obtained from Henan Borun New Materials Co., Ltd., Zhengzhou, China. This compound is identified as a white powder with a modulus of 2.0, containing 25% to 29% sodium oxide (Na_2_O) and 49.0% to 54.0% silicon dioxide (SiO_2_). The particle size distribution indicates that 98.9% of the material passes through a 100-mesh sieve, and it exhibits a dissolution time of 59 s. The critical oxides are SiO_2_ and Na_2_O. SiO_2_ serves as the fundamental silicon source necessary for synthesizing C-(A)-S-H gel, while Na_2_O increases the system’s alkalinity, thereby facilitating the activation of precursors. Furthermore, its adjustable modulus enables optimal alkali activation within the composite system.

(2) NaOH

The sodium hydroxide used in this study was obtained from Tianjin Aopusheng Chemical Co., Ltd., Tianjin, China, with a purity of ≥96%. It appears as white granules. It effectively increases the system’s pH to facilitate the dissolution of aluminosilicates, works synergistically with sodium silicate to regulate the alkali modulus, and enhances the rate of hydration during alkali activation.

### 2.2. Methods

**Figure 1:**
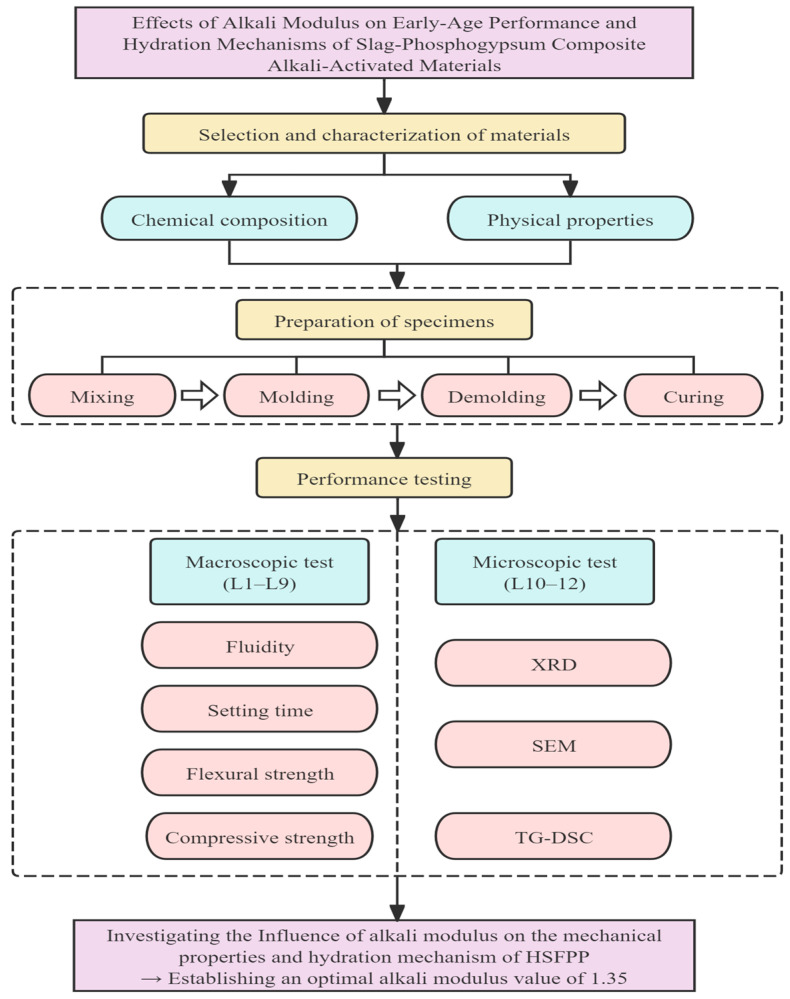
The illustration of the methodology development.

Figure 1 illustrates a schematic overview of the methodologies utilized to conduct both macroscopic and microscopic testing.

For sample preparation, an alkaline activator was initially formulated by mixing Na_2_SiO_3_, NaOH, and water using a magnetic stirrer. Subsequently, slag, fly ash, phosphogypsum, and Ca(OH)_2_ were combined with the alkaline activator and stirred for a duration of three minutes. The resulting slurry was then poured into specimen molds, which were subjected to vibration to eliminate entrapped air bubbles. The surface of the molds was subsequently leveled using a scraper. After a curing period of six hours within the molds, the specimens were demolded and transferred to a curing chamber, where they were maintained until reaching the designated curing age. The curing process was conducted in an environment maintained at a temperature of 20 °C ± 2 °C and a relative humidity exceeding 90%.

#### 2.2.1. Macroscopic Tests

The setting time tests, fluidity tests, and strength tests were all conducted in accordance with the procedures specified in the “Test Methods of Cement and Concrete for Highway Engineering (JTG3420-2020)” [35]. The fluidity tests and setting time tests were implemented immediately following the preparation of the HSFP. The strength tests were carried out at designated curing intervals of 3, 7, and 28 days, under controlled curing conditions maintained at a temperature of 20 °C ± 2 °C and relative humidity exceeding 90%.

#### 2.2.2. Microscopic Tests

The SEM analysis was performed utilizing the Thermo Scientific Apreo S HiVac (Waltham, MA, USA), a high-resolution field emission scanning electron microscope (FE-SEM) featuring high-vacuum operation for secondary electron imaging and capable of achieving an ultra-low voltage resolution of 0.9 nm at an accelerating voltage of 500 V. Correspondingly, the XRD analysis was conducted employing the same Thermo Scientific Apreo S HiVac FE-SEM system, which offers high-vacuum conditions for secondary electron imaging and maintains an ultra-low voltage resolution of 0.9 nm at 500 V. Furthermore, TG-DSC measurements were carried out using this identical FE-SEM apparatus, distinguished by its high-vacuum functionality and ultra-low voltage resolution of 0.9 nm at an accelerating voltage of 500 V.

## 3. Analysis of Physical and Mechanical Properties

HSFP was synthesized using sodium hydroxide and sodium silicate as alkaline activators. This investigation examines the effect of the alkali modulus on the mechanical properties of HSFP. Specimens were fabricated under varying alkaline conditions and subjected to curing protocols in accordance with established standards. Critical technical parameters, including flexural strength, compressive strength, fluidity, and setting time, were systematically evaluated.

### 3.1. Testing Program

The mix design employed in the experiments detailed in this chapter, as presented in Table 7 and Table 8, comprises nine distinct groups. These groups are characterized by a water-to-binder ratio of 0.54 and a slag powder proportion of 69%. The alkaline environment is controlled by varying the alkali modulus.

### 3.2. Effect of Alkali Modulus on Early Performance of HSFP

#### 3.2.1. Effect of Alkali Modulus on Strength

Table 9 presents the effect of activator dosage on the flexural and compressive strengths of the specimens at various curing ages. The table denotes the average value as AVG and the standard deviation as SD, using these abbreviations consistently in the subsequent tables.

**Figure 2:**
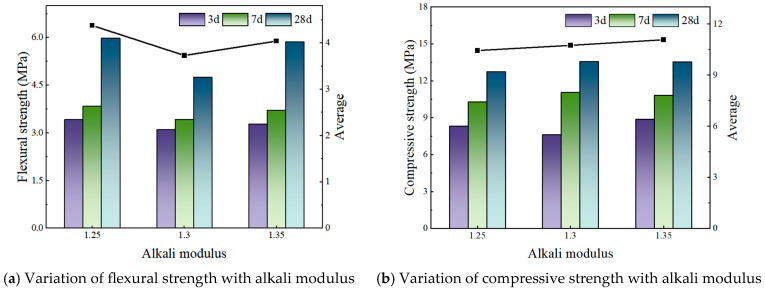
FA:PG is 4:6—Strength under different alkali modulus.

**Figure 3:**
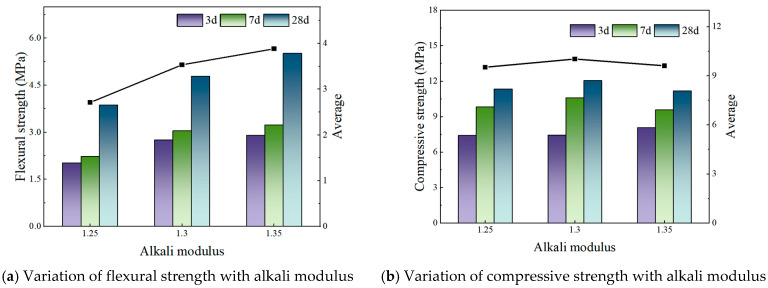
FA:PG is 1:9—Strength under different alkali modulus.

**Figure 4:**
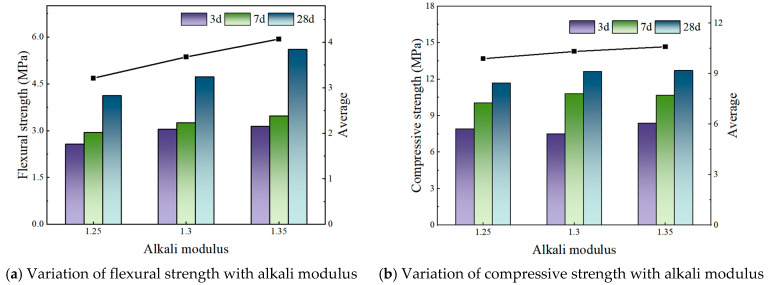
FA:PG is 1:5—Strength under different alkali modulus.

Figure 2, Figure 3 and Figure 4 illustrate the effect of alkali modulus on the flexural and compressive strengths of concrete at different curing ages.

Figure 2 and Figure 3 demonstrate that variations in the fly ash to phosphogypsum (FA:PG) ratio significantly affect the relationship between alkali modulus and the resulting mechanical properties. Specifically, at an FA:PG ratio of 4:6 with a constant alkali content of 3.1%, the flexural strength exhibits a non-monotonic response to increasing alkali modulus, initially decreasing and subsequently increasing. Notably, the flexural strength at an alkali modulus of 1.35 (5.87 MPa) is marginally lower than that observed at 1.25 (5.13 MPa). In contrast, compressive strength increases linearly with alkali modulus, although the rate of increase is relatively modest.

At an FA:PG ratio of 1:9, maintaining the alkali content at 3.1%, flexural strength shows a positive correlation with alkali modulus, indicating a marked enhancement. Specifically, the flexural strength at an alkali modulus of 1.35 is 1.51 times greater than that at 1.25. Conversely, compressive strength initially increases with alkali modulus, reaching a maximum at 1.3, followed by a slight decline; however, these changes are comparatively minor.

For an FA:PG ratio of 1:5 with a fixed alkali content of 3.1%, flexural strength increases linearly as the alkali modulus rises. Similarly, compressive strength initially increases, peaking at an alkali modulus of 1.3, before experiencing a modest decrease, with overall variations remaining relatively small.

#### 3.2.2. Effect of Alkali Modulus on the Flow Time

Effects of the alkali modulus on flow time are shown in Table 10.

When maintaining a constant phosphogypsum-to-slag powder ratio, variations in the alkali modulus exhibit minimal effect, as all recorded flow times consistently fall within the optimal interval of 10 to 14 s. The majority of the observed discrepancies in these measurements can be attributed to experimental error.

#### 3.2.3. Effect of Alkali Modulus on the Setting Time

Table 11 illustrates the effect of the activator quantity of activator on the setting time.

**Figure 5:**
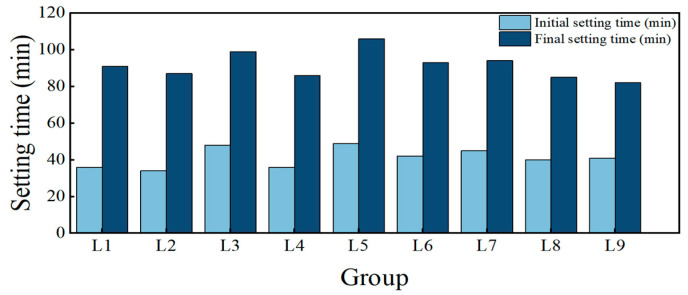
Effect of alkali modulus on setting time of HSFP.

Figure 5 illustrates the effects of alkali modulus on setting time.

As illustrated in Table 11 and Figure 5, variations in alkali modulus have a negligible effect on the setting time.

## 4. Effect of Alkali Modulus on the Hydration Mechanism of HSFP

### 4.1. Testing Program

As outlined in Section 3, the compressive strength obtained at an alkali modulus of 1.25 is slightly greater than that recorded at 1.35; however, the alkali modulus of 1.25 requires a higher consumption of NaOH. Therefore, the current investigation focuses on the hydration mechanism at an alkali modulus of 1.35. To this purpose, three experimental groups, designated L10 through L12, were established to examine the effect of varying alkali modulus on the hydration mechanism of HSFP. The quantities of activator incorporated in the HSFP mix designs are detailed in Table 12 and Table 13.

In this section, to provide a more comprehensive elucidation of the effect exerted by the phosphogypsum-to-slag powder ratio on the hydration mechanism of HSFP, six novel mix designs, different from those presented in Section 3, were developed. Subsequently, the technical properties of these newly formulated mixtures were systematically evaluated.

(1) Strength

**Figure 6:**
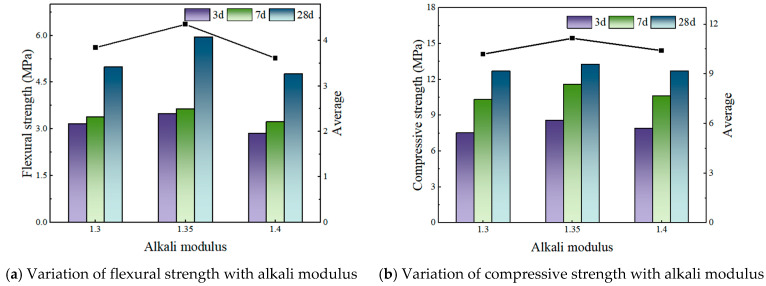
The effect of alkali modulus on the strength of HSFP.

The effect of alkali modulus on the strength of HSFP is shown in Table 14 and Figure 6.

(i) Figure 6a,b present the analysis of samples L10 through L12, conducted at a fixed alkali content of 3.0%, to assess the effect of changing the alkali modulus within the range of 1.3 to 1.4.

(ii) For the sample with an alkali modulus of 1.3 (L10), the strength development was comparatively the weakest. This result is primarily due to the high alkalinity of the activator combined with an insufficient supply of soluble silica under these conditions. Although the high concentration of hydroxide ions (OH^−^) promotes the rapid breakdown of the slag’s glassy phase, thus accelerating early-ion dissolution, the deficiency of silicate ions limits their effectiveness as nucleation and precipitation sites. Consequently, the hydration products mainly form a loosely structured, low-polymerization C-(A)-S-H gel. Additionally, the excessively fast reaction rate has an adverse effect on the long-term stability of AFt, which in turn impairs the mechanical strength.

(iii) When the alkali modulus was increased to 1.4 (L12), the strength performance improved compared to L10 but still remained inferior to that of L11. In this case, the activator provided a sufficient amount of soluble silicate species; however, the relatively lower concentration of OH^−^ ions reduced the alkali activation driving force within the system. Consequently, the overall degree of hydration was limited, thereby preventing the macroscopic strength from reaching its maximum potential.

(iv) At an intermediate alkali modulus of 1.35 (L11), the strength reached its peak value, indicating the existence of an optimal alkali modulus range for this system. At this specific modulus, the alkalinity (OH^−^ concentration) and the soluble silica content (SiO_2_) provided by the activator are synergistically balanced. Adequate alkalinity ensures the continuous and effective dissolution of gel components, while sufficient silicate ions promote the orderly precipitation of hydration products. This synergy leads to the formation of a large quantity of highly polymerized C-(A)-S-H gels with superior mechanical properties. These gels, interwoven with an appropriate amount of AFt crystals, collectively form the densest microstructure observed.

(2) Fluidity and setting time

**Figure 7:**
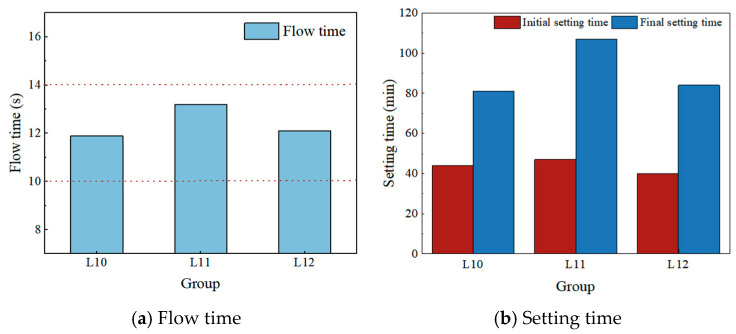
The effect of alkali modulus on the flow time and setting time.

The effects of alkali modulus on the flow time and setting time of HSFP are shown in Table 15 and Figure 7.

As shown in Table 15 and Figure 7, and consistent with the findings presented in Section 3, variations in alkali modulus do not significantly affect fluidity or setting time. Its effect is primarily determined by the amounts of slag powder, phosphogypsum, and fly ash.

### 4.2. Effect of Alkali Modulus on Hydration Mechanism of HSFP

#### 4.2.1. XRD

**Figure 8:**
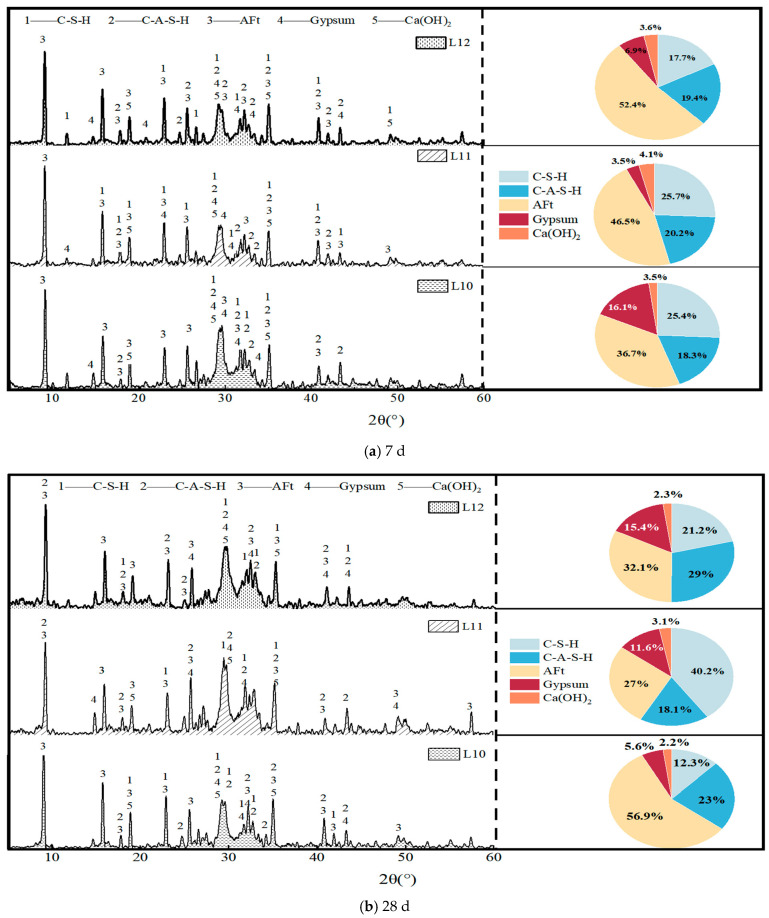
XRD analysis diagram under alkaline modulus change.

The XRD results at 7 and 28 days for the L10-L12 groups (with varying alkali modulus) are shown in Figure 8.

To comprehensively elucidate the fundamental mechanism through which the alkali modulus affects macroscopic strength, this research investigates a semi-quantitative XRD analysis to characterize the composition of hydration products at curing intervals of 7 and 28 days across varying alkali modulus ratios (L10 to L12).

(1) At 7 days of age:

(i) In the L10 group, which is characterized by an alkali modulus of 1.3, the hydration product distribution reflected a system featuring high alkalinity but limited silicon availability. The data showed that the AFt content was the lowest among all groups, standing at 37.2%, while the gypsum residue was the highest, measuring 16.3%. These findings imply that although the elevated initial alkalinity effectively activates the cementitious components, the insufficient reactive silicon hinders both the stable nucleation and growth of AFt and the proper formation of C-(A)-S-H gel, which was recorded at 44.2%. The substantial amount of unreacted gypsum (16.3%) indicates an incomplete reaction process, leading to a porous microstructure within the final hydration products and consequently resulting in the lowest macroscopic strength observed.

(ii) In the L12 group, with an alkali modulus of 1.4, the hydration product distribution changed towards conditions marked by reduced alkalinity and increased silica content. This group demonstrated the highest AFt formation, reaching 52.4%. However, the development of C-(A)-S-H gel was significantly inhibited, with its content dropping to a minimum of 37.1%. This outcome can be ascribed to the relatively weak alkaline environment, which is inadequate to fully and continuously dissolve the active silicon and aluminum phases present in the slag. Consequently, there is an overall shortage of ions available for hydration reactions. Under these conditions, the limited ions preferentially promote AFt formation, while the generation of C-(A)-S-H gel—the primary matrix contributing to strength—is restricted due to the scarcity of reactive species. Although the AFt crystal framework is well-established, the lack of a dense gel matrix to fill and bind the structure prevents the attainment of optimal macroscopic strength.

(iii) The L11 group, defined by an alkali modulus of 1.35, displayed a well-balanced synergistic state in its hydration product profile. This group produced a moderate yet sufficient amount of AFt (46.5%) and, notably, achieved the highest proportion of C-(A)-S-H gel (45.9%) among the three groups examined. Furthermore, the gypsum residue was minimal (3.5%), indicating the highest degree of hydration and the most efficient utilization of reactive materials. At this optimal alkali modulus, the alkalinity provided by the activator is ideally matched to the silica source; the appropriate alkalinity promotes the continuous dissolution of cementitious components and stable AFt formation, while the adequate silica content supports the development of a high-strength, dense C-(A)-S-H gel matrix.

**Figure 9:**
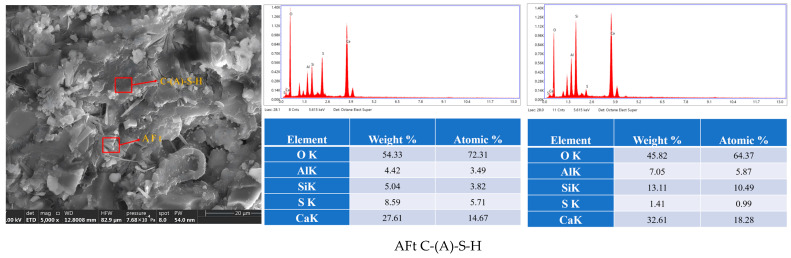
Transmission electron microscopy image of AFt and gel after 7 days.

Figure 9 presents transmission electron microscopy images of AFt and gel samples after 7 days. In contrast to oxide analysis, which reflects overall bulk chemical composition, elemental analysis precisely characterizes localized elemental distribution within specific phases. This approach helps verify the formation of hydration products.

(2) At 28 days of age

(i) Within the L10 group, defined by an alkali modulus of 1.3, a distinctive developmental trajectory was identified at 28 days. Notably, the content of AFt exhibited a significant increase, attaining the highest proportion among the three groups at 56.9%, while gypsum was substantially depleted, remaining at a mere 5.6%. This observation implies that the highly alkaline environment associated with this specific mix ratio sustains activation over time, facilitating the continuous release of aluminum phases during prolonged curing and thereby promoting ongoing AFt formation. Conversely, the content of C-(A)-S-H gel in this group was the lowest, measured at 35.3%, suggesting that the primary limitation arises from an insufficient silicon supply due to the low-modulus activator. This disparity, characterized by enhanced crystallinity coupled with reduced gel content despite an overall higher degree of reaction, impedes the development of a dense microstructure. Consequently, this limitation adversely affects the macroscopic mechanical strength and may contribute to issues related to volumetric stability.

**Figure 10:**
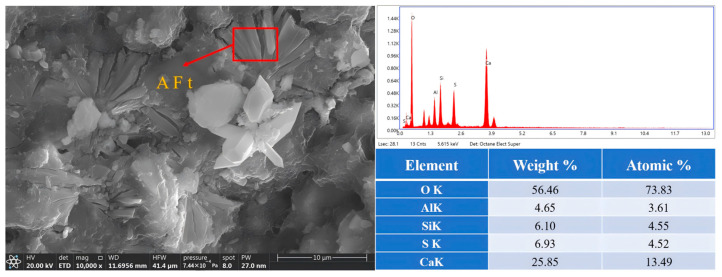
Transmission electron microscopy image of AFt after 28 days.

Figure 10 presents transmission electron microscopy images of AFt after 28 days.

(ii) In the L12 group, which is characterized by an alkali modulus of 1.4, the long-term evolution showed a contrasting trend. Compared with the measurements at 7 days, the content of AFt significantly decreased from 52.4% to 32.1%, while the amount of residual gypsum did not decrease but rather increased to 15.4%. This observation strongly indicates that the initial alkalinity of the system was insufficient, leading to the early consumption of activation energy. Consequently, it was difficult to maintain a high level of chemical reactivity in the long term. The subsequent decrease in pH likely caused the transformation or dissolution of some of the initially formed AFt. Meanwhile, due to the depletion of the alkaline driving force, the abundant silicon source was not effectively used for additional gel formation. This resulted in a large amount of unreacted gypsum and restricted the overall reaction progress.

(iii) The L11 group, with an alkali modulus of 1.35, showed an optimal distribution of hydration products at 28 days, indicating enhanced reaction synergy and system stability. This group had the highest proportion of C-(A)-S-H gel (58.2%) while maintaining a moderate AFt content and a relatively high gypsum conversion rate, with only 11.6% gypsum remaining. These results imply that at this specific alkali modulus, the system reached a dynamic equilibrium between alkali activation and silica availability. This balance prevented the insufficient gel formation caused by silica deficiency observed in the L10 group and alleviated the premature decline in reaction activity due to inadequate alkali content seen in the L12 group. As a result, a stable, comprehensive, and efficient long-term hydration process was established, producing a composite microstructure composed of a high-strength C-(A)-S-H gel matrix reinforced by an appropriate amount of stable AFt crystals. This microstructural configuration macroscopically corresponded to the superior mechanical performance observed.

#### 4.2.2. TG-DSC

**Figure 11:**
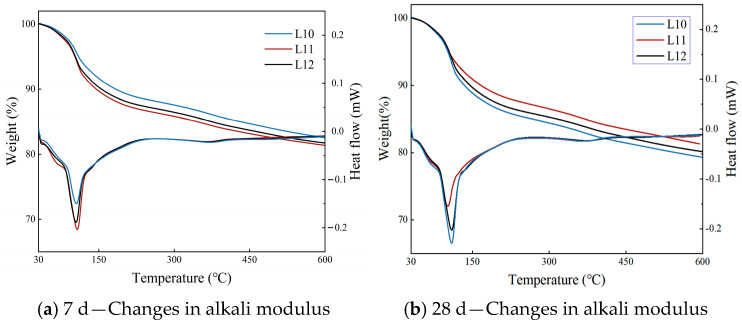
Different alkali moduli under TG-DSC conditions.

The TG-DSC curves at different curing ages and alkali modulus are shown in Figure 11.

**Figure 12:**
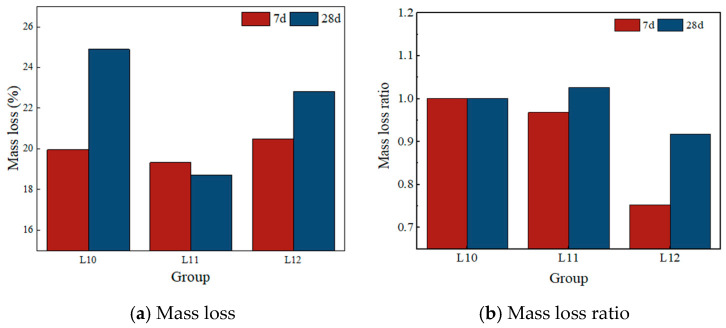
Test mass loss and mass loss ratio.

Table 16, along with Figure 12, presents the mass loss values and the corresponding mass loss ratios across different alkali modulus levels. In Table 17, the mass loss ratios are calculated using Group L10 as the reference point; specifically, the mass loss recorded at each time interval for each experimental group is normalized by dividing it by the mass loss observed in Group L10 at the same age. Figure 12 illustrates both the absolute mass loss data and the derived mass loss ratios. It is important to note that Groups L10, L11, and L12 represent distinct alkali modulus values of 1.3, 1.35, and 1.4, respectively.

The observed pattern of variation in the alkali modulus is as follows:

(i) In Group L10, which is characterized by an alkali modulus of 1.3, the total mass loss increased significantly from 19.955% at 7 days to 24.887% at 28 days. This trend indicates that the hydration reaction within this system remains highly active but incomplete during the critical curing period between 7 and 28 days. The pronounced increase in AFt content, as revealed by XRD analysis, suggests that the observed mass loss is primarily driven by the high-alkali environment, which promotes the continued hydration of alunite and the conversion of a substantial portion of free water into crystallization water. Consequently, this process results in an elevated rate of chemically bound water loss. These findings confirm that the formulation with a low alkali modulus, due to insufficient effective silicon sources, experiences impaired and unbalanced early hydration reactions, ultimately leading to suboptimal mechanical properties.

(ii) For Group L12, with an alkali modulus of 1.4, the mass loss gradually increased from 20.483% at 7 days to 22.815% at 28 days, reflecting a steady progression of the hydration process. Compared to Group L10, this increase is mainly attributed to the slow formation of C-(A)-S-H gel, as confirmed by XRD analysis. This behavior is ascribed to the relatively low initial alkalinity inherent in the high-modulus formulation, which results in weak chemical activation. Consequently, the reactive ions within the cementitious components are not fully or rapidly released, leading to a deceleration of the overall reaction rate. This sluggish reaction impedes the accumulation of hydration products during later stages, which is macroscopically manifested as a limited potential for strength development.

(iii) In Group L11, characterized by an alkali modulus of 1.35, the mass loss slightly decreased from 19.321% at 7 days to 18.719% at 28 days. This pattern suggests high hydration efficiency and the early completion of the primary hydration reactions within this mixture. By day 7, the principal chemical reactions had essentially concluded, with the subsequent period up to 28 days primarily involving the refinement and stabilization of the microstructure of hydration products. This stage entailed a minor release of bound water, corresponding to the gradual decrease in mass loss. The combination of rapid initial reaction kinetics and subsequent structural stabilization underpins the superior mechanical performance observed in Group L11.

Thermogravimetric analysis provided additional evidence supporting the beneficial effect of the alkali modulus on the total volume of hydration products. Notably, in Group L11, which achieved optimal mechanical strength at an alkali modulus of 1.35, the mass loss either stabilized or slightly decreased by 28 days. This suggests that the hydration reaction had effectively reached completion during the early stages. Subsequent developments were characterized primarily by the microstructural refinement of the hydration products, leading to the establishment of a stable and high-strength matrix. In contrast, Groups L10 (alkali modulus 1.3) and L12 (alkali modulus 1.4) exhibited a continuous and marked increase in mass loss throughout the 28-day period, indicating slower and less balanced hydration kinetics. X-ray diffraction analysis identified that the mass increase in Group L10 was predominantly attributable to the extensive late-stage formation of AFt, whereas in Group L12, it corresponded to the gradual evolution of gel phases. Both groups failed to develop synergistic product structures during the initial hydration phase, resulting in inefficient compensatory formation of hydration products and, consequently, limited strength gains during the later stages.

Table 17 presents the effects of alkali modulus on the heat flux curve and the corresponding area of HSFP.

The observed pattern of variation in the alkali modulus is as follows:

(i) In Group L10, characterized by an alkali modulus of 1.3, TG-DSC analysis reveals a notable increase in the characteristic decomposition peak temperature, rising from 105.32 °C at 7 days to 109.31 °C at28 days. This thermal shift is accompanied by an increase in peak heat flux intensity and an expansion of the peak area from 9.66 to 10.08. These observations suggest the formation of hydration products with enhanced crystallinity and improved thermal stability, primarily AFt, during the later stages of hydration. This interpretation is corroborated by a significant rise in AFt content as determined by XRD analysis. The data imply that although the initially elevated alkalinity associated with the low alkali modulus formulation inhibited early AFt formation, it subsequently facilitated gradual crystallization and optimal growth of AFt over time. Nevertheless, despite the improved quality of AFt formed at a delayed stage, its integration with the relatively weak and insufficiently abundant C-(A)-S-H gel matrix was inadequate, resulting in suboptimal macroscopic mechanical properties.

(ii) For Group L12, defined by an alkali modulus of 1.4, kinetic analysis indicates a shift in the characteristic peak temperature from 104.59 °C to 109.41 °C over a 28-day period, while the peak area remains essentially constant, varying slightly from 9.76 to 9.78. The observed temperature increase suggests structural stabilization and maturation of the early-formed hydration products. Concurrently, the plateau in peak area implies that the chemical reactions associated with AFt formation reach equilibrium after 7 days. These findings indicate that the high alkali modulus formulation, due to insufficient initial alkalinity, leads to premature depletion of reactive species, thereby impeding the sustained progression of hydration reactions. Consequently, this limitation results in a restricted reaction extent and an inadequate driving force for further intensification of the reaction process.

(iii) In Group L11, with an alkali modulus of 1.35, the characteristic peak temperature decreases significantly from 107.13 °C to 102.32 °C, accompanied by a reduction in peak area from 9.79 to 8.14. This trend reflects a late-stage hydration process predominantly governed by structural optimization. The observed decreases in temperature and peak area should not be interpreted as indicative of reaction degradation; rather, these changes are attributed to microscopic restructuring phenomena, including densification of the gel structure within the hydration product matrix.

At seven days, the peak area ratio followed the order L11 > L12 > L10, indicating that the efficiency of early-stage AFt formation was highest under the optimal modulus condition (L11). In contrast, the low modulus group (L10) exhibited the lowest yield, likely due to a deficiency in silicon sources and pronounced alkali inhibition. By 28 days, this trend reversed, with the sequence becoming L10 > L12 > L11. This inversion is attributed to the sustained high-alkali environment in the L10 group, which promoted compensatory AFt formation during the later stages. The L12 group experienced stagnation, presumably due to insufficient initial alkalinity, while the L11 and L10 groups demonstrated a reduction in total AFt content as a result of system maturation. This observed pattern closely corresponds with findings from alkali content experiments, collectively suggesting that optimizing macroscopic strength—whether through adjustments in content or modulus—reflects a chemical equilibrium that prioritizes efficient early-stage reactions and late-stage structural stability over the maximization of product quantity.

## 5. Conclusions

This study primarily investigates the effect of alkali modulus on the early-age properties and hydration mechanisms of the HSFP. The main findings are summarized as follows:

The effect of alkali modulus on the strength of the HSFP is significantly affected by the fly ash–phosphogypsum (FA:PG) ratio. At an FA:PG ratio of 4:6, the flexural strength initially decreases and then increases as the alkali modulus values increase, while the compressive strength shows a consistent upward trend. In contrast, at FA:PG ratios of 1:5 and 1:9, the flexural strength increases linearly with the alkali modulus, whereas the compressive strength first rises and then experiences a slight decline. The alkali modulus has minimal effect on fluidity and setting time.

A reduced alkali modulus results in insufficient formation of C-(A)-S-H gel attributable to a deficiency of silicon, which in turn produces a porous microstructure primarily consisting of AFt. In contrast, an elevated alkali modulus hinders reaction kinetics and leads to significant retention of gypsum, as unreacted particles become prematurely encapsulated by hydration products. Both scenarios negatively affect the mechanical properties.

An optimal alkali modulus value of 1.35 has been identified, at which the balance between alkalinity and the concentration of available soluble silica promotes complete hydration. The highest content of C-(A)-S-H gel is observed at 28 days.

## Figures and Tables

**Table 1:** Chemical composition of slag powder.

Chemical Composition	CaO	SiO_2_	Al_2_O_3_	SO_3_	Fe_2_O_3_	MgO
Content (%)	34.00	34.50	17.70	1.64	1.03	6.01

**Table 2:** Physical properties of slag powder.

Index	Specific Surface Area (m^2^/kg)	Flow Ratio (%)	Density (g/cm^3^)	Loss on Ignition (%)	Water Content (%)
Indicator	429.00	98.00	3.10	0.84	0.45

**Table 3:** Chemical composition of fly ash.

Chemical Composition	SiO_2_	Al_2_O_3_	Fe_2_O_3_	CaO	MgO	K_2_O
Content (%)	40.00	30.00	4.20	10.00	2.50	1.10

**Table 4:** Physical properties of fly ash.

Index	Density (g/m^3^)	Stacking Density (g/m^3^)	Specific Surface Area (g/m^2^)	Water Absorption (%)	Water Requirement (%)	Water Content (%)
Indicator	2.10	0.79	3500	106	≤100%	≤0.9%

**Table 5:** Chemical composition of phosphogypsum.

Chemical Composition	CaO	MgO	SO_3_	Al_2_O_3_	Fe_2_O_3_	SiO_2_	P_2_O_5_	F
Content (%)	32.14	0.4	43.38	0.18	0.04	9.45	1.18	0.80

**Table 6:** Chemical composition of Ca(OH)_2_.

Chemical Composition	CaO	SiO_2_	Al_2_O_3_	Ca (OH) _2_	Fe_2_O_3_	MgO	CaCO_3_
Content (%)	73.53	0.78	0.597	89.51	0.128	0.68	3.98

**Table 7:** The effect of the amount of the activator on the mix ratio of HSFP.

Group	Slag Powder	Fly Ash: Phosphogypsum	Alkali Content	Alkali Modulus
L1	69%	4:6	3.1%	1.25
L2				1.3
L3				1.35
L4		1:9		1.25
L5				1.3
L6				1.35
L7		1:5		1.25
L8				1.3
L9				1.35

**Table 8:** The detailed mixing ratio of the accelerator dosage to HSFP.

Group	Slag Powder (g)	Fly Ash (g)	Phosphogypsum (g)	Ca(OH)_2_ (g)	Na_2_SiO_3_ (g)	NaOH (g)	H_2_O (g)
L1	1167.5	203	304.6	108	133.2	28.4	1059.3
L2	1167.5	203	304.6	108	138.4	26.4	1061
L3	1167.5	203	304.6	108	144.0	24.8	1063.2
L4	1167.5	50.8	456.8	108	133.2	28.4	1059.3
L5	1167.5	50.8	456.8	108	138.4	26.4	1061
L6	1167.5	50.8	456.8	108	144.0	24.8	1063.2
L7	1167.5	86.3	421.3	108	133.2	28.4	1059.3
L8	1167.5	86.3	421.3	108	138.4	26.4	1061
L9	1167.5	86.3	421.3	108	144.0	24.8	1063.2

**Table 9:** The effect of the amount of activator on the strength of HSFP.

Group	Flexural Strength (MPa)						Compressive Strength (MPa)					
	3-Day		7-Day		28-Day		3-Day		7-Day		28-Day	
	AVG	SD	AVG	SD	AVG	SD	AVG	SD	AVG	SD	AVG	SD
L1	3.42	0.11	3.84	0.21	5.99	0.11	8.32	0.21	10.28	0.52	12.74	0.19
L2	3.11	0.16	3.43	0.23	4.75	0.16	7.63	0.36	11.05	0.36	13.56	0.35
L3	3.28	0.17	3.71	0.21	5.87	0.23	8.87	0.41	10.82	0.46	13.54	0.21
L4	2.03	0.16	2.23	0.19	3.87	0.25	7.40	0.51	9.82	0.29	11.34	0.61
L5	2.77	0.21	3.05	0.27	4.79	0.31	7.43	0.25	10.59	0.46	12.05	0.27
L6	2.90	0.21	3.23	0.20	5.52	0.29	8.07	0.38	9.57	0.36	11.18	0.38
L7	2.57	0.11	2.94	0.16	4.12	0.35	7.91	0.41	10.05	0.45	11.69	0.29
L8	3.05	0.18	3.25	0.24	4.73	0.17	7.49	0.39	10.81	0.19	12.63	0.34
L9	3.14	0.21	3.47	0.22	5.60	0.33	8.38	0.42	10.67	0.37	12.72	0.46

**Table 10:** The fluidity of HSFP under different concentrations of excipients.

Group	Flow Time (s)	
	AVG	SD
L1	13.8	0.25
L2	11.8	0.36
L3	14.4	0.21
L4	14.3	0.15
L5	12.6	0.11
L6	11.3	0.35
L7	13.5	0.41
L8	13.0	0.22
L9	12.9	0.32

**Table 11:** Results of setting time of HSFP.

Group	Initial Setting Time (min)		Final Setting Time (min)	
	AVG	SD	AVG	SD
L1	36	2.0	91	3.2
L2	34	2.2	87	2.1
L3	48	1.6	99	1.0
L4	36	1.8	86	3.6
L5	49	2.2	106	2.8
L6	42	2.1	93	2.6
L7	45	1.6	94	1.8
L8	40	1.5	85	3.0
L9	41	2.4	82	2.2

**Table 12:** The effect of alkali modulus on the mix ratio of HSFP.

Group	Slag Powder	Fly Ash–Phosphogypsum	Alkali Content	Alkali Modulus
L10	69%	1:5	3.00%	1.3
L11				1.35
L12				1.4

**Table 13:** The detailed mixing ratio of the accelerator dosage to HSFP.

Number	Slag Powder (g)	Fly Ash (g)	Phosphogypsum (g)	Ca(OH)_2_ (g)	Na_2_SiO_3_ (g)	NaOH (g)	H_2_O (g)
L10	1167.5	86.3	421.3	108.0	134.0	25.6	1058.2
L11	1167.5	86.3	421.3	108.0	139.2	23.6	1059.9
L12	1167.5	86.3	421.3	108.0	144.8	22.0	1062.1

**Table 14:** Results of the strength of HSFP.

Group	Flexural Strength (MPa)						Compressive Strength (MPa)					
	3-Day		7-Day		28-Day		3-Day		7-Day		28-Day	
	AVG	SD	AVG	SD	AVG	SD	AVG	SD	AVG	SD	AVG	SD
L10	3.17	0.21	3.39	0.21	4.99	0.23	7.53	0.21	10.34	0.56	12.71	0.32
	0.10	0.02	0.12	0.006	0.18	0.01	0.39	0.01	0.26	0.02	0.36	0.02
L11	3.49	0.11	3.64	0.32	5.95	0.33	8.6	0.32	11.59	0.33	13.27	0.34
	0.21	0.01	0.19	0.01	0.32	0.02	0.16	0.01	0.25	0.02	0.41	0.03
L12	2.85	0.21	3.23	0.12	4.77	0.21	7.91	0.31	10.62	0.23	12.69	0.62
	0.09	0.004	0.25	0.02	0.17	0.005	0.39	0.01	0.26	0.01	0.31	0.02

**Table 15:** The effects of alkali modulus on the setting time of HSFP.

Group	Initial Setting Time (min)		Final Setting Time (min)		Flow Time (s)	
	AVG	SD	AVG	SD	AVG	SD
L10	44	0.24	81	0.52	11.9	0.36
L11	47	0.25	107	0.28	13.2	0.19
L12	40	0.24	84	0.62	12.1	0.41

**Table 16:** The effect of alkali modulus on the mass loss of HSFP.

Group	Mass Loss		Mass Loss Ratio	
	7 d	28 d	7 d	28 d
L10	19.955%	24.887%	1.000	1.000
L11	19.321%	18.719%	0.968	0.752
L12	20.483%	22.815%	1.026	0.917

**Table 17:** The effect of alkali modulus changes on the characteristics of endothermic peak in heat flow curve.

Group	Temperature (°C)		Heat Flow (mW)		Heat Transfer Area (J/g)	
	7 d	28 d	7 d	28 d	7 d	28 d
L10	105.32	109.31	−0.149969	−0.229013	9.66451	10.07693
L11	107.13	102.32	−0.203676	−0.154704	9.78669	8.13952
L12	104.59	109.41	−0.188702	−0.202755	9.76365	9.78079

## Data Availability

The original contributions presented in this study are included in the article. Further inquiries can be directed to the corresponding author.
